# Uroplakin II Expression in Breast Carcinomas Showing Apocrine Differentiation: Putting Some Emphasis on Invasive Pleomorphic Lobular Carcinoma as a Potential Mimic of Urothelial Carcinoma at Metastatic Sites

**DOI:** 10.1155/2016/2940496

**Published:** 2016-05-26

**Authors:** Shogo Tajima, Kenji Koda

**Affiliations:** Department of Pathology, Fujieda Municipal General Hospital, Shizuoka 426-8677, Japan

## Abstract

Uroplakin II antibody is exclusively specific for urothelial carcinoma. Nonurothelial carcinoma has not been reported to be immunoreactive for uroplakin II. In the present study, we hypothesized that breast carcinoma showing apocrine differentiation, such as invasive pleomorphic lobular carcinoma (IPLC) and apocrine carcinoma (AC), stains positive for uroplakin II. We identified 6 cases of IPLC between 2000 and 2014 by searching a computerized pathological database. We randomly selected 10 cases of each classic invasive lobular carcinoma (cILC) and AC and five cases of apocrine metaplasia (AM) that coexisted in a surgically resected breast carcinoma specimen. Immunohistochemistry was performed for uroplakin II, GATA3, CK7, CK20, and other representative markers positive for urothelial carcinoma. All cases of IPLC, AC, and AM, except those of cILC, showed immunoreactivity for uroplakin II. Poorly differentiated urothelial carcinoma sometimes shows similar morphology to IPLC with the following immunophenotype: CK7+, CK20−, GATA3+, and uroplakin II+. In the present study, this immunophenotype was observed in all the cases of IPLC and AC. Therefore, when studying metastatic, poorly differentiated carcinoma showing the aforementioned immunophenotype, we should consider the possibility of it being IPLC in addition to metastatic urothelial carcinoma.

## 1. Introduction

Recently developed uroplakin II antibody (clone: BC21) was found to be exclusively specific to the urothelium and urothelial carcinomas when evaluated in various normal and neoplastic tissues [1]. It specifically stained urothelium among 37 US Food and Drug Administration normal tissue types, including 3 breast tissue samples [1]. Regarding neoplastic tissue, 20 tumor types were immunostained with it and urothelial carcinoma was exclusively positive for it, except for one of eighty-eight prostatic adenocarcinomas. However, this one positive case was considered to be metastatic urothelial carcinoma that had spread to the prostate gland [1].

GATA3 is an immensely sensitive and relatively specific marker for urothelial as well as breast carcinoma [2]. When encountering metastatic carcinoma that is positive for GATA3, the possibility of urothelial and breast carcinoma should be first considered. If, in addition to GATA3, uroplakin II is positive for metastatic carcinoma, it should be considered as metastatic urothelial carcinoma, as has been suggested by previous studies; to the best of our knowledge, no breast carcinoma has ever been reported to be positive for uroplakin II in the English language literature [1, 3, 4].

We happened to examine a case of invasive pleomorphic lobular carcinoma (IPLC) metastatic to the urinary bladder as routine surgical pathology practice. Since it resembled a poorly differentiated urothelial carcinoma, immunohistochemistry was performed with markers positive for urothelial carcinoma, such as uroplakin II, GATA3, p63, p40, and 34*β*E12 [4, 5]. This case was found to be diffusely positive for these markers except for p63 and p40, which showed focal positivity.

IPLC is a subtype of invasive lobular carcinoma (ILC), which exhibited relatively high-grade nuclei and abundant cytoplasm compared with classic invasive lobular carcinoma (cILC); IPLC is considered to behave more aggressively than cILC [6]. In addition, IPLC exhibits apocrine differentiation [7–9]. Thus, we hypothesized that apocrine differentiation might be related to uroplakin II immunoreactivity.

Examining metastatic carcinoma with poorly differentiated and high-grade morphology and GATA3 expression, breast carcinoma including IPLC and poorly differentiated urothelial carcinoma are considered for the differential diagnosis. Among breast carcinomas showing apocrine differentiation, apocrine carcinoma (AC) is not recognized as exhibiting poorly differentiated morphology. Thus, the aim of this study was to examine uroplakin II expression in IPLC. Moreover, immunohistochemical analyses of other markers expected to be positive for urothelial carcinoma are included in this study. We further analyzed cases of cILC and apocrine metaplasia (AM) for comparison.

## 2. Materials and Methods

We identified 58 surgically resected cases of ILC between 2000 and 2014 from a computerized pathological database. On the basis of histological examination results, we classified the cases exhibiting enlarged nuclei (approximately 4 times the size of a lymphocyte) with an irregular nuclear membrane, marked nuclear hyperchromasia, prominent nucleoli, increased mitotic activity, and moderate-to-abundant eosinophilic, finely granular cytoplasm as IPLC, in addition to the loosely cohesive growth pattern and immunonegativity for E-cadherin commonly noted in ILC [10]. Six cases (6/58, 10.3%) fulfilled the criteria of IPLC. Ten surgically resected cases of cILC and AC and five cases of AM coexisting in a surgically resected specimen of breast carcinoma were randomly selected. Formalin-fixed, paraffin-embedded blocks were available for all the selected cases. Each operative specimen was fixed in 10% buffered-formalin followed by paraffin embedding.

Immunohistochemistry was performed for 4 *μ*m thick sections obtained from paraffin-embedded blocks using primary antibodies against uroplakin II, GATA3, p63, p40, CK7, CK20, 34*β*E12, and gross cystic disease fluid protein-15 (GCDFP-15), respectively. Additionally, all the selected cases of IPLC and cILC were immunostained with E-cadherin to confirm the diagnosis. The detailed information of these antibodies was summarized in Table 1. Immunohistochemistry was performed using a BenchMark GX Autoimmune Stainer and an I-View DAB Detection Kit. The interpretations were scored as follows: 0 = less than 5% tumor cell positivity; +1 = 5–10% tumor cell positivity; +2 = 11–50% tumor cell positivity; and +3 = more than 50% tumor cell positivity.

This study was approved by the institutional review board.

## 3. Results

**Figure 1:**
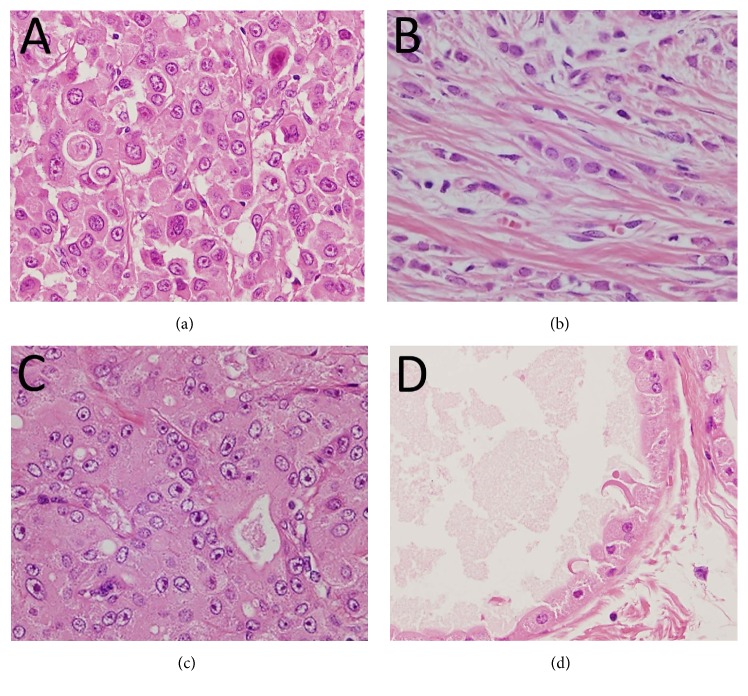
Histological findings. (a) IPLC case 2: nuclear enlargement and eosinophilic cytoplasm observed in tumor cells (×400). (b) cILC case 2: nuclear enlargement of the tumor cells is modest compared with that of IPLC. Reduced cytoplasm in the tumor cells is observed (×400). (c) AC case 2: cytoplasmic abundance and eosinophilia of the tumor cells are more prominent than those of IPLC (×400). (d) AM case 3: the cytoplasmic abundance and eosinophilia of the constituent cells are conspicuous but the nuclei are less atypical than those of IPLC and AC (×400). IPLC: invasive pleomorphic lobular carcinoma; cILC: classic invasive lobular carcinoma; AC: apocrine carcinoma; AM: apocrine metaplasia.

Representative cases of IPLC and cILC were shown in Figures 1(a) and 1(b), respectively. AC and AM exhibited a more prominent cytoplasmic abundance and eosinophilia than IPLC with characteristic cytoplasmic granularity, but the nuclei were more atypical in AC than in AM (Figures 1(c) and 1(d)).

Selected IPLC and cILC cases were confirmed to be immunonegative for E-cadherin. Except for E-cadherin, the result of immunohistochemical analyses of each case is shown in Table 2 and a summary of immunoreactivity for each immunohistochemical marker is presented in Table 3.

**Figure 2:**
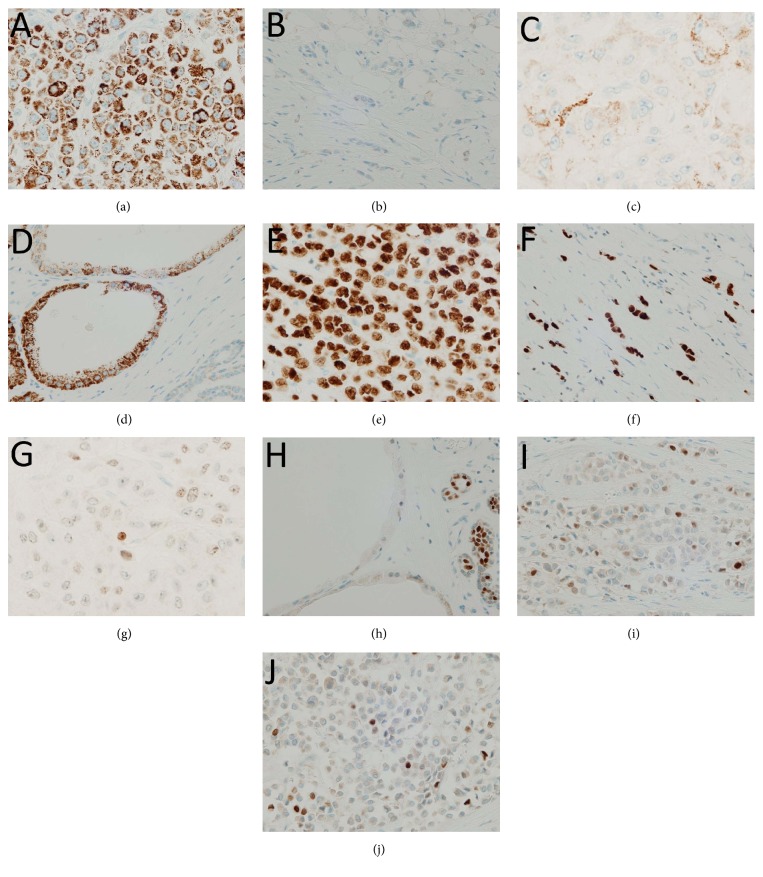
Immunohistochemical findings. (a) IPLC case 3: immunopositivity for uroplakin II (score 3+) (×400). (b) cILC case 4: immunonegativity for uroplakin II (score 0) (×400). (c) AC case 4: immunopositivity for uroplakin II (score 2+) (×400). (d) AM case 3: immunopositivity for uroplakin II (score 3+). Note immunonegativity for normal breast epithelium (×400). (e) IPLC case 5: immunopositivity for GATA3 (score 3+) (×400). (f) cILC case 3: immunopositivity for GATA3 (score 3+) (×400). (g) AC case 8: immunopositivity for GATA3 (score 1+) (×400). (h) AM case 4: immunonegativity for GATA3 (score 0). Note immunopositivity for normal breast epithelium (×400). (i) IPLC case 3: scattered immunopositive tumor cells for p63 (×400). (j) IPLC case 3: scattered immunopositive tumor cells for p40 (×400). IPLC: invasive pleomorphic lobular carcinoma; cILC: classic invasive lobular carcinoma; AC: apocrine carcinoma; AM: apocrine metaplasia.

Uroplakin II immunoreactivity for IPLC, cILC, AC, and AM is shown by a representative figure of each tumor type (Figures 2(a)–2(d)). Immunopositivity of IPLC, AC, and AM to uroplakin II was observed in all cases examined; they shared considerable immunoreactivity for GCDFP-15 as a common feature. Prominence of uroplakin II expression was in the following order: AM, IPLC, and AC. All the cases of AM expressed uroplakin II with score 3+; however, half (3 of 6 and 5 of 10, resp.) of IPLC and AC showed uroplakin II expression of score 3+ with the rest of the cases exhibiting its expression of score 1+ or 2+; and cILC was immunonegative for uroplakin II in all cases examined. GATA3 immunoreactivity for IPLC, cILC, AC, and AM is shown by a representative figure of each tumor type (Figures 2(e)–2(h)). GATA3 was expressed in all cases of IPLC and cILC with almost all the cases being evaluated as score 3+, but its expression status seemed to be lower in AC with 5 of 10 cases (50%) being evaluated as score 1+ or 2+. GATA3 was less likely to be expressed in AM (1/5: 20%, score 1+). Immunopositivity for p63 was observed only in IPLC (Figure 2(i)) and cILC (2/6: 33% and 1/10: 10%, resp.) with a score of 1+ in all the positive cases. p40 showed low expression in IPLC (Figure 2(j)) and cILC when compared to p63 (2/6: 33% and 1/10: 10%, resp.) and no expression in AC and AM. CK7 was immunopositive with a score of 3+ for all cases examined. However, CK20 was completely immunonegative. Furthermore, expression of 34*β*E12 was observed exclusively in IPLC and cILC with a constant 3+ score. GCDFP-15 was consistently immunoreactive for IPLC, AC, and AM cases with a score of 3+, while it had a variable immunoreactivity in cILC (ranging from 0 to 2+). A score of 2+ was observed in only one case (10%) and a score of 1+ in 6 cases (60%) with the remaining 3 cases (30%) showing immunonegativity.

## 4. Discussion

We discovered that IPLC, AC, and AM had similar immunophenotypes considering uroplakin II and GCDFP-15 immunoreactivity. Uroplakin II is one of the four isoforms (others are uroplakins Ia, Ib, and III), which enhances the permeability barrier and strength of the urothelium and is normally produced by urothelial cells [11]. Likewise, GCDFP-15 is a marker of apocrine differentiation [12]. Previous studies have shown that breast carcinoma is immunonegative for uroplakin II [1, 3, 4], in which breast carcinoma showing apocrine differentiation was probably not included. In addition, since cILC examined in this study was immunonegative for uroplakin II, the interpretation that apocrine differentiation and uroplakin II immunopositivity are correlated seems to be valid.

Considering the viewpoint of differential diagnosis, the distinction between IPLC and poorly differentiated urothelial carcinoma seems to be difficult when encountering GATA3-expressing poorly differentiated metastatic carcinoma. Decrease in E-cadherin expression is noted in poorly differentiated urothelial carcinoma [13], and a loss of expression is a feature of the plasmacytoid variant of urothelial carcinoma, which is similar to ILC and expresses GATA3 [5]. Even though IPLC shows apocrine differentiation, its degree of cytoplasmic abundance and eosinophilic granularity does not match that of AC and AM. However, distinction between AC and urothelial carcinoma is relatively easy at the metastatic site, since there are no reports that urothelial carcinoma has eosinophilic granular cytoplasm as prominent as that observed in AC. We thus focused on the distinction between IPLC and poorly differentiated urothelial carcinoma.

In addition to total immunoreactivity for 34*β*E12, in some cases, IPLC expressed p63 and p40. The expression of these three markers is typically observed in urothelial carcinoma [4, 5]. When encountering metastatic carcinoma, CK7 and CK20 are often stained to identify the primary site of it [14, 15]. According to previous reports as well as our present findings, CK7 is expected to be commonly expressed in IPLC and urothelial carcinoma [5]. On the other hand, CK20 is not expressed in IPLC and is often expressed in urothelial carcinoma [5]. Thus, if CK20 immunoreactivity is observed in metastatic carcinoma in addition to GATA3, the primary site of the metastatic carcinoma is expected to be the urinary bladder. However, CK20 is not a marker commonly immunopositive for urothelial carcinoma, and its expression decreases when urothelial carcinoma is poorly differentiated [5]. On the other hand, CK7 is more frequently expressed than CK20 [5]. Immunopositivity for GATA3 is maintained in poorly differentiated urothelial carcinoma [16]; uroplakin II is expressed in approximately 60% high-grade urothelial carcinoma [1, 17]. Thus, metastatic urothelial carcinoma showing poorly differentiated morphology with CK7+, CK20−, GATA3+, and uroplakin II+ is anticipated. In addition, metastatic IPLC is expected to show CK7+, CK20−, GATA3+, and uroplakin II+ immunophenotype. Therefore, when encountering metastatic carcinoma with poorly differentiated morphology that shows this immunophenotype, the possibility of IPLC should also be considered even though uroplakin II is immunopositive.

In conclusion, this study is the first to report the immunoreactivity of nonurothelial carcinomas for uroplakin II. These are breast carcinomas showing apocrine differentiation, such as IPLC and AC. At the metastatic site, IPLC and poorly differentiated urothelial carcinoma might show similar morphologies, which indicated that uroplakin II immunopositivity in metastatic carcinoma with poorly differentiated morphology does not directly lead to the diagnosis of poorly differentiated urothelial carcinoma and the possibility of IPLC should also be considered.

## Figures and Tables

**Table 1:** The detailed information of the antibodies used in the present study.

Antibodies to	Clone	Dilution	Pretreatment	Source
Uroplakin II	BC21	1 : 100	HIER	Biocare Medical, Concord, CA
GATA3	L50-823	1 : 100	HIER	Biocare Medical, Concord, CA
p40	BC28	1 : 100	HIER	Biocare Medical, Concord, CA
p63	4A4	1 : 100	HIER	Biocare Medical, Concord, CA
CK7	OV-TL 12/30	1 : 100	HIER	Dako, Glostrup, Denmark
CK20	Ks20.8	1 : 100	HIER	Dako, Glostrup, Denmark
34*β*E12	34*β*E12	1 : 100	HIER	Dako, Glostrup, Denmark
GCDFP-15	23A3	1 : 200	HIER	Dako, Glostrup, Denmark
E-cadherin	NCH-38	1 : 100	HIER	Dako, Glostrup, Denmark

GCDFP-15: gross cystic disease fluid protein-15; HIER: heat-induced epitope retrieval.

**Table 2:** Results showing immunohistochemical analysis of each case of IPLC, cILC, AC, and AM.

Case	Uroplakin II	GATA3	p63	p40	CK7	CK20	34*β*E12	GCDFP-15
IPLC								
1	3+	3+	0	0	3+	0	3+	3+
2	2+	3+	0	0	3+	0	3+	3+
3	3+	2+	1+	1+	3+	0	3+	3+
4	2+	3+	0	0	3+	0	3+	3+
5	3+	3+	1+	1+	3+	0	3+	3+
6	1+	3+	0	0	3+	0	3+	3+
cILC								
1	0	3+	0	0	3+	0	3+	1+
2	0	3+	0	0	3+	0	3+	2+
3	0	3+	0	0	3+	0	3+	1+
4	0	3+	0	0	3+	0	3+	0
5	0	3+	0	0	3+	0	3+	1+
6	0	3+	0	0	3+	0	3+	1+
7	0	3+	1+	1+	3+	0	3+	1+
8	0	3+	0	0	3+	0	3+	0
9	0	3+	0	0	3+	0	3+	1+
10	0	3+	0	0	3+	0	3+	0
AC								
1	2+	3+	0	0	3+	0	0	3+
2	3+	3+	0	0	3+	0	0	3+
3	1+	2+	0	0	3+	0	0	3+
4	2+	2+	0	0	3+	0	0	3+
5	3+	3+	0	0	3+	0	0	3+
6	3+	3+	0	0	3+	0	0	3+
7	1+	2+	0	0	3+	0	0	3+
8	1+	1+	0	0	3+	0	0	3+
9	3+	1+	0	0	3+	0	0	3+
10	3+	3+	0	0	3+	0	0	3+
AM								
1	3+	0	0	0	3+	0	0	3+
2	3+	1+	0	0	3+	0	0	3+
3	3+	0	0	0	3+	0	0	3+
4	3+	0	0	0	3+	0	0	3+
5	3+	0	0	0	3+	0	0	3+

IPLC: invasive pleomorphic lobular carcinoma; cILC: classic invasive lobular carcinoma; AC: apocrine carcinoma; AM: apocrine metaplasia.

**Table 3:** Summary of immunoreactivity of IPLC, cILC, AC, and AM for each immunohistochemical marker used in this study.

Score	Uroplakin II	GATA3	p63	p40	CK7	CK20	34*β*E12	GCDFP-15
IPLC (*n* = 6)								
3+	3 (50%)	5 (83%)	0	0	21 (100%)	0	21 (100%)	21 (100%)
2+	2 (33%)	1 (17%)	0	0	0	0	0	0
1+	1 (17%)	0	2 (33%)	2 (33%)	0	0	0	0
0	0	0	4 (67%)	4 (67%)	0	21 (100%)	0	0
cILC (*n* = 10)								
3+	0	10 (100%)	0	0	10 (100%)	0	10 (100%)	0
2+	0	0	0	0	0	0	0	1 (10%)
1+	0	0	1 (10%)	1 (10%)	0	0	0	6 (60%)
0	10 (100%)	0	9 (90%)	9 (90%)	0	10 (100%)	0	3 (30%)
AC (*n* = 10)								
3+	5 (50%)	5 (50%)	0	0	10 (100%)	0	0	10 (100%)
2+	2 (20%)	3 (30%)	0	0	0	0	0	0
1+	3 (30%)	2 (20%)	0	0	0	0	0	0
0	0	0	10 (100%)	10 (100%)	0	10 (100%)	10 (100%)	0
AM (*n* = 5)								
3+	5 (100%)	0	0	0	5 (100%)	0	0	5 (100%)
2+	0	0	0	0	0	0	0	0
1+	0	1 (20%)	0	0	0	0	0	0
0	0	4 (80%)	5 (100%)	5 (100%)	0	5 (100%)	5 (100%)	0

IPLC: invasive pleomorphic lobular carcinoma; cILC: classic invasive lobular carcinoma; AC: apocrine carcinoma; AM: apocrine metaplasia.
